# Considerations upon Otorrhœa, Particularly in Children, and upon a New Method of Treatment

**Published:** 1868-01

**Authors:** M. Bonnafont

**Affiliations:** Corresponding Member of the Academy


### CONSIDERATIONS UPON OTORRHŒA, PARTICULARLY IN CHILDREN, AND UPON A NEW METHOD OF TREATMENT.

Communicated to the Imperial Academy of Sciences, April, 1867.

BY M. BONNAFONT, Corresponding Member of the Academy.

Translated from l'Union Medicale of July 2d, 1867.

All persons are not equally predisposed to this affection; in general, we observe it most frequently in constitutions that are lymphatic, strumous, gouty, etc. There are some exceptions to this rule; thus the affections of the ear are often developed after a cutaneous eruption, as scarlatina and rubeola, more particularly after the last, without our being able to give the reasons for this preference.

The age at which this kind of otorrhœa ordinarily manifests itself is from six to ten years, sometimes sooner, but rarely later; it is at this time, therefore, that we should hasten to direct an energetic treatment against the disease, for the simplest piece of negligence, on account of the susceptibility and delicacy of the organs of hearing, may allow the most serious lesions to encroach upon this apparatus. At this age, indeed, it is not the deafness alone that is to be dreaded, but even dumbness as the inevitable result of the loss of hearing. Nearly one-third of the children who are found in the establishments for the deaf and dumb, both in France and in foreign countries, owe their infirmity to nothing but the destruction of the apparatus of the middle ear, by neglected otorrhœas; while it is probable that if these children had been subjected to suitable treatment at the proper time, it would have been successful, at least in a large number of them, in arresting the progress of the disease, and in preventing thus an infirmity henceforth incurable, which must ever cause grief to the parents' heart.

*Prognosis.*—The prognosis of this affection is, we think, according to the extent or the seat of the lesion; thus the inflammation may occupy the whole surface of the canal, and it may present less of gravity than if it occupied a position even the most limited, in the neighborhood of the tympanum; in the first case the ear may be for a long time diseased without inducing any disorder of the hearing, while, in the second, it is rare that the tympanic membrane, whether by the continued contact with pus, or by the extension of the inflammation, does not finish by injuring itself, and by compromising, later on, the function of the organ. Besides, another circumstance which renders these ulcerations at the bottom of the canal very much more serious than those which are developed in the regions nearer to the meatus, is this: we know that the glands which secrete the cerumen do not extend beyond the external two-thirds of the canal, and that beyond, the flesh is extremely slight, very red, very sensitive, and applied almost directly upon the bone, from which it is separated only by a very thin layer of cellular tissue. It follows, from this anatomical disposition, of the greatest importance in auricular pathology, that all that portion of the canal which is provided with glandular tissues may be for a long period diseased, without the subjacent bone being affected; while, in a region lower down, the slightest ulceration of the skin attacks, pretty soon, the periosteum and the bone, if we do not promptly arrest its progress. * *

*Treatment.*—The first indication to be fulfilled consists in making a careful examination of the canal, in order to ascertain the seat of disease and the degree of its extent. But in general, when we are consulted, it is seldom that the patients, large or small, have not the canal obstructed with matter; it is on this account that we must devote three or four days to these preliminary cases, consisting in cleansing perfectly the canal and in freeing it from all the matters which may conceal the ulcerations; it is for this purpose that I recommend the patient to take, three or four times a-day, ear baths of poppy water, then to make with the same liquid, injections, pretty strong, so that the liquid, in returning upon itself, may bring with it all the foreign matters. * * * * *

So long as there is no suppuration, it is less essential that the injections penetrate into the interior, but the case is different when pus is being thrown out from an ulcerated surface, especially if deeply situated. We can easily understand that if, while the meatus is obstructed by the engorgement of tissues, the suppuration accumulating in the lowest portions of the canal, will compress the tympanum, will cause its laceration, and later on, its destruction, as well as that of the apparatus of the little bones.

It is to avoid a similar accident, that I have employed for many years small dilating canulas of caoutchouc. Whatever may be the narrowing of the canal, we may always cause a little sound, previously coated with cerate, to glide in; and when this has been introduced, we can easily cause others of larger size to penetrate. But before replacing a sound by another, we should take advantage of the opening already made to use injections, and to relieve, as much as possible, the bottom of the canal of the purulent matters which may be found there. * * When the discharge resists our endeavors and threatens to pass into the chronic form, the local treatment should be conducted in the most energetic manner, and by a succession of the means I have indicated. We must always commence by satisfying ourselves, by the use of the otoscope, of the region which the lesions occupy and whence the pus proceeds; when we have recognized the diseased point, we should at once cauterize with a small crayon of nit. silver, such as I use. These little cauterizations, made with care, cause very little, if any pain, and can be repeated every second day.

In the interval we may use astringent and styptic injections, with acetate of lead, of the strength of 1 gramme to 100 grammes of water, sulphate of zinc of the same strength, or, which is excellent, sulphate of alumina, of the strength of 2, 4, and even 6 grammes to the 100 grammes of the liquid. This last is what I most frequently employ, especially since, having tried it in the hospitals in gonorrhœa, it has given me very satisfactory results. * * * * *

When this malady appears in a strumous subject, or in a lymphatic constitution, it is very evident that, in this case, we should unite the local treatment with an internal medication, the energy and activity of which must be proportioned to the degree of the lymphatic character of the individual. The local treatment, without being neglected, should be conducted with prudence, and should follow the modifications produced by the constitutional treatment. If, on the contrary, otorrhœa be engrafted upon a sanguineous constitution, M. Kramer counsels, with reason, that we should not attend to the general condition, but should treat the case by purely local means. * *

In order that medication should be applied in a rational manner, it is necessary to see the parts affected; for it is not an indifferent thing to cauterize healthy tissue, the tympanum particularly. It is for the purpose of facilitating this examination for a large number of physicians, that I have caused to be made a novel otoscope, very simple, which does not require the assistance of any lamp, and whose shape renders it very portable. This instrument will be found of equally felicitous application in the examination of other organic lesions, such as those of the neck of the uterus, etc. * * *

This instrument has the great advantage of only occupying one hand, of allowing every kind of inclination to be given to the light, and of illuminating the bottom of canals very obliquely situated, by directing into it a very intense luminous ray.

This otoscope is composed of two tubes: the vertical one, which serves as a handle, contains a small wax candle, such as is used to light up the little altars in the month of May. This tube is pierced at the bottom with many apertures, in order to allow the passage of air, which is necessary to nourish the light. The other tube, forming the principal body of the instrument, presents at its superior part a large opening corresponding to the axis of the vertical tube, and by which the flame of the candle escapes. Its posterior extremity is guarded by a small reflecting mirror in platinum, and the anterior by a bi-convex lens, the power of refraction of which has been calculated so as to make a very great concentration of the flame at the greatest possible distance, in order to fulfil the two following conditions: 1. To cause the greatest possible light to penetrate to the bottom of the auditory canal, notwithstanding its narrowness; 2. Then to leave between the illuminated point and the instrument sufficient space to allow not only of seeing well, but also, with the other hand, to perform in the canal or on the tympanum any operation that may be deemed necessary. This tube may be lengthened or shortened, in order to give to the luminous ray, a greater or less concentration, according to the cavities we desire to illumine.

The tubes may be taken apart, and one made to inclose the other, the instrument then is very compact, and very portable. —*Southern Journal of Medical Sciences.*

